# Characteristics of gay and bisexual men who rarely use HIV risk reduction strategies during condomless anal intercourse: Results from the FLUX national online cohort study

**DOI:** 10.1371/journal.pone.0233922

**Published:** 2020-06-01

**Authors:** Johann Kolstee, Martin Holt, Jeff Jin, Mohamed A. Hammoud, Louisa Degenhardt, Lisa Maher, Toby Lea, Garrett Prestage

**Affiliations:** 1 Kirby Institute, UNSW Sydney, Kensington, NSW, Australia; 2 Centre for Social Research in Health, UNSW Sydney, Kensington, NSW, Australia; 3 National Drug and Alcohol Research Centre, UNSW Sydney, Kensington, NSW, Australia; 4 Burnet Institute, Melbourne, Victoria, Australia; 5 German Institute for Addiction and Prevention Research, Catholic University of Applied Sciences, Cologne, Germany; Ohio State University, UNITED STATES

## Abstract

**Purpose:**

To understand the characteristics of a minority of Australian gay and bisexual men (GBM) who, despite an increase in the number and availability of HIV risk reduction strategies, do not consistently use a strategy to protect themselves from HIV.

**Methods:**

This analysis is based on data from 2,920 participants in a national, online, prospective observational cohort study. GBM who never or rarely used HIV risk reduction strategies (NRR) were compared with two groups using multivariate logistic regression: i) GBM using pre-exposure prophylaxis (PrEP) and ii) GBM frequently using risk reduction strategies (FRR) other than PrEP.

**Results:**

Compared to PrEP users, NRR men were younger (p<0.0001), less socially engaged with gay men (p<0.0001) and less likely to have completed a postgraduate (p<0.05) or undergraduate degree (p<0.05). They were also less likely to have recently used amyl nitrite (p<0.05), erectile dysfunction medication (p<0.05) and cocaine (p<0.05) in the previous 6 months. Compared with FRR men, NRR men were less likely to have completed a postgraduate (p<0.0001) or undergraduate degree (p<0.05), scored higher on the sexual sensation-seeking scale (p<0.0001) and were more likely to identify as versatile (p<0.05), a bottom (p<0.05) or very much a bottom (p<0.05) during anal sex.

**Conclusions:**

NRR men were largely similar to other Australian GBM. However, our analysis suggests it may be appropriate to focus HIV prevention interventions on younger, less socially engaged and less educated GBM, as well as men who prefer receptive anal intercourse to promote the use of effective HIV risk reduction strategies.

## Introduction

Sex between men remains the most common way that HIV is transmitted in Australia [1]. In 2017, 70.1% of the 1013 new diagnoses of HIV in Australia were attributed to sex between men [1]. There are a range of strategies to prevent HIV, including condoms, the use of antiretroviral drugs as biomedical prevention, and behavioural risk reduction (such as serosorting and strategic positioning). These strategies differ in their effectiveness [2–5] as well as the length of time over which they have been used by or available to gay and bisexual men (GBM) in Australia.

Data from national behavioural surveillance surveys of Australian GBM in 2013 indicated that most men (98.7%) frequently use at least one strategy to prevent HIV when having anal sex with casual partners [6]. This research was conducted before the widespread promotion of use of undetectable viral load prior to sex or HIV treatment as prevention (TasP), and access to PrEP. In this research, HIV-negative men who had condomless anal intercourse with casual partners most commonly used serosorting (46.9%) followed by condoms (40.5%) [6]. HIV-positive men in Australia most commonly used TasP (58.4%) followed by serosorting (55.4%) [6].

Recent Australian national and state HIV strategies and community-based education campaigns have encouraged the use of effective HIV risk reduction strategies (condoms, PrEP, and TasP) while also reinforcing the importance of communication between partners [7–9]. These strategies and campaigns mirror the understandings of risk and acceptable prevention strategies among GBM [10–11]. They have supported the awareness, acceptability and access to effective HIV risk reduction strategies in Australia.

Effective antiretroviral treatment (ART) for HIV has been available since the mid-1990s and early initiation to achieve viral suppression is highly effective in preventing transmission [5]. Recent data indicated that HIV treatment uptake and viral suppression are high among HIV-positive Australian GBM [12]. The use of antiretroviral medications for prevention (pre-exposure prophylaxis, PrEP) by HIV-negative people has also been shown to be highly effective [13]. First available to GBM in Australia through personal importation (pre-2014), then large research trials (2014–2018) [14–15], PrEP is now widely available and publicly subsidized [16].

Condoms have been the main preventative tool for HIV transmission for GBM since the beginning of the HIV epidemic [17]. Serosorting, restricting sex to partners believed to be the same HIV status, has also been widely used by GBM [17]. Strategic positioning is another strategy which involves partners taking the insertive or receptive position during condomless anal intercourse, depending on the HIV status of one’s partner [18]. This strategy is based on the understanding that HIV negative men are less likely to acquire HIV when taking the insertive rather than the receptive position during condomless anal intercourse with an HIV positive man [19]. Receptive condomless anal intercourse is the main route of HIV infection among GBM [19].

Despite a range of effective prevention tools, many GBM recently diagnosed with HIV report receptive condomless anal intercourse with another man prior to their diagnosis, and limited evidence of risk reduction [20]. Inconsistent or infrequent use of HIV risk reduction increases the chance that GBM may have condomless anal intercourse with someone with undiagnosed HIV infection, a key driver of new HIV infections in Australia [21].

In national behavioural surveillance surveys, GBM have been found to use condoms consistently at higher rates than their heterosexual counterparts [12]. Consistent condom use has, however, fallen from 46% in 2013 to 31% in 2017 among GBM having anal intercourse with casual partners in Sydney and Melbourne [22]. This fall has occurred in the context of increasing PrEP uptake nationally among GBM having anal intercourse with casual partners (1% in 2015 to 16% in 2017) [23]. While confidence in the capacity to discuss condoms with partners remains high, particularly among HIV negative GBM (66.3%), few HIV negative GBM (6.8%) report having positive experiences using condoms [24].

Drug use has been shown to impact HIV sexual risk and vice versa [25]. However, research into GBM using methamphetamine and erectile dysfunction medications in Australia has indicated increasing concurrent PrEP use [26]. Drug use and the contexts and cultures in which it occurs should also be considered when attempting to support GBM adopt HIV risk reduction strategies.

In the context of increasing access to, and use of, PrEP, growing prevention optimism may contribute to GBM being increasingly inclined to forego condom use [27]. Prevention optimism is the reduction in the use of HIV prevention strategies (such as condoms) due to the belief that other people in a sexual network are using effective strategies (such as PrEP or TasP). In 2017, nearly a quarter of Australian GBM (23%) were found to be less concerned about HIV and believed that condomless anal intercourse was safer because other people were using PrEP, even though they were not using PrEP themselves [28].

Despite advances in HIV prevention, some GBM appear to remain “unprotected” and at risk of HIV, as they do not appear to consistently use any HIV risk reduction strategy. Understanding the characteristics and practices of these GBM may help inform targeted HIV prevention campaigns to increase the effectiveness and coverage of HIV prevention. For a comprehensive HIV prevention response factors at the individual, interpersonal, community, institutional and structural levels should be understood and addressed [29]. Using data from a large cohort study of Australian GBM, we analysed the characteristics of GBM who engaged in condomless anal intercourse and did not consistently practice HIV risk reduction in comparison to GBM who frequently used one or more risk reduction strategies.

## Methods

This analysis is based on data from the Following Lives Undergoing Change (Flux) study, which is a national online prospective observational study of drug use among GBM in Australia. The study protocol has been described in detail elsewhere [30]. In summary, recruitment into the study occurred from 2014 onwards via key gay community social media, websites, sexual networking websites and mobile phone applications. Online questionnaires were completed at baseline and then repeatedly at six-month intervals. Participant consent was obtained online at the start of the questionnaire. Compensation was not provided for participation. All procedures performed in this study were in accordance with the National Statement on Ethical Conduct in Human Research (National Health and Medical Research Council, Australia) and with the 1964 Helsinki Declaration and its later amendments or comparable ethical standards. Ethical approval for this study was provided by the Human Research Ethics Committee of UNSW Sydney (reference number: HC14075).

### Participants

Participants were eligible to participate in this study if they identified as male, identified as gay or bisexual or reported having sex with another man in the previous year, lived in Australia and were at least sixteen years of age. A total of 2,920 participants from across Australia enrolled into the study between 2014 and 2017 and fulfilled the minimum data requirements for the online questionnaire.

### Measures

The demographic items included age, education, employment status, sexual orientation, country of birth and ethnicity. All the demographic variables were used as they appear in Table 1, except for ethnicity where responses were merged into either Anglo-Australian or other background. Age was operationalised as a continuous variable, and education, employment status, country of birth and sexual orientation as categorical variables. Participants self-reported their HIV status with HIV positive GBM also reporting whether they were taking anti-retroviral treatment. PrEP use or non-use was reported by HIV negative and status unknown GBM.

**Table 1:** Participant characteristics of GBM in the FLUX cohort 2014–2017.

Variable	Total (n = 2920)	Frequent Risk Reduction (FRR) (n = 2252)	No Risk Reduction (NRR) (n = 253)	PrEP users (n = 415)
**Mean Age** (S.D.)	35.4 (13.0)	34.7 (12.9)	32.4 (13.3)	41.2 (11.8)
**Education Level**		970 (43.2%)		
Less than university	1,264 (43.3%)	714 (31.8%)	151 (59.7%)	143 (34.5%)
Undergrad completed	915 (31.3%)	562 (25.0%)	62 (24.5%)	139 (33.5%)
Postgrad completed	735 (25.17%)		40 (15.8%)	133 (32.0%)
Did not answer	6 (0.2%)			
**Ethnicity/Cultural Background**		1,637 (73.0%)	179 (71.6%)	
Anglo-Australian	2,108 (72.2%)	604 (27.0%)	71 (28.4%)	292 (70.4%)
Other backgrounds	798 (27.3%)			123 (29.6%)
Did not answer	14 (0.5%)			
**Sexual Orientation**				
Gay/Homosexual	2,609 (89.4%)	1,994 (88.5%)	224 (88.5%)	391 (94.2%)
Bisexual	250 (8.6%)	210 (9.3%)	24 (9.5%)	16 (3.9%)
Heterosexual	4 (0.1%)	2 (0.1%)	2 (0.8%)	0 (0%)
Other	57 (2.0%)	46 (2.0%)	3 (1.2%)	8 (1.9%)
**Sexual Sensation Seeking Scale**–Mean Score (S.D.)	30.0 (6.3)	29.1 (6.2)	32.4 (6.3)	32.7(5.6)
**Gay Social Engagement Scale**–Mean Score (S.D.)	3.6 (1.6)	3.5 (1.6)	3.4 (1.6)	4.5 (1.5)
**Drug use (monthly or more frequently)**				
Amyl nitrite	616 (21.1%)	368 (16.3%)	64 (25.3%)	184 (44.3%)
Cocaine	69 (2.4%)	48 (2.1%)	1 (0.4%)	20 (4.8%)
EDM	395 (13.5%)	231 (10.3%)	30 (11.9%)	134 (32.3%)
GHB	87 (3.0%)	39 (1.7%)	8 (3.2%)	40 (9.7%)
Crystal Methamphetamine	171 (5.9%)	107 (4.8%)	17 (6.7%)	47 (11.3%)
**HIV Status**				
HIV-positive	196 (6.7%)	190 (8.4%)	6 (2.4%)	0 (0%)
HIV-negative	2295 (78.6%)	1727 (76.7%)	161 (63.6%)	407 (98.1%)
Do not know/unsure	429 (14.7%)	335 (14.9%)	86 (34.0%)	8 (1.9%)
**HIV Testing (Ever tested)**				
Never tested	391 (13.4%)	312 (13.9%)	77 (30.4%)	2 (0.5%)
Yes	2,517 (86.2%)	1929 (85.7%)	175 (69.2%)	413 (99.5%)
Did not answer	12 (0.4%)			
**Top versus Bottom Identity**			5 (2.0%)	
Very much a top	137 (4.7%)	11 (0.5%)	32 (12.7%)	21 (5.1%)
Top	530 (18.2%)	426 (18.9%)	114 (45.4%)	72 (17.3%)
Versatile	1,366 (46.8%)	1,056 (46.9%)	75 (29.9%)	196 (47.2%)
Bottom	703 (24.1%)	532 (23.6%)	25 (10.0%)	96 (23.1%)
Very much a bottom	175 (6.0%)	120 (5.3%)		30 (7.2%)
Did not answer	9 (0.3%)			
**Sex with CASUAL partners** (among HIV negative)				
(Last 6 months)	590 (20.8%)	529 (24.3%)		
Condoms only	345 (12.1%)	29 (1.3%)	22 (8.7%)	39 (9.4%)
PrEP	167 (5.9%)	163 (7.5%)	0 (0%)	316 (76.1%)
Insertive condomless—no PrEP	130 (4.6%)	107 (4.9%)	4 (1.6%)	0 (0%)
Receptive condomless withdrawal—no PrEP	232 (8.2%)	156 (7.2%)	23 (9.1%)	0 (0%)
Receptive condomless ejaculation–no PrEP			76 (30.0%)	0 (0%)
**Sex with CASUAL partners** (among HIV positive)				
(Last 6 months)	23 (0.8%)	23 (1.1%)		
Condoms only	42 (1.5%)	42 (1.9%)	0 (0%)	0 (0%)
Undetectable and treated	65 (2.3%)	65 (3.0%)	0 (0%)	0 (0%)
Receptive condomless only–no ARV	3 (0.1%)	2 (0.1%)	0 (0%)	0 (0%)
Insertive comdomless withdrawal–no ARV	8 (0.3%)	8 (0.4%)	1 (0.4%)	0 (0%)
Insertive condomless ejaculation–no ARV			5 (2.0%)	0 (0%)
**Sex with CASUAL partners** (among HIV unknown)				
(Last 6 months)	81 (2.9%)	*(*		
Condoms only	23 (0.8%)	72 (3.3%)	8 (3.2%)	1 (0.2%)
Insertive condomless—no PrEP	23 (0.8%)	14 (0.6%)	7 (2.8%)	2 (0.5%)
Receptive condomless withdrawal—no PrEP	55 (1.9%)	19 (0.9%)	4 (1.6%)	0 (0%)
Receptive condomless ejaculation–no PrEP		25 (1.1%)	26 (10.3%)	4 (1.0%)
**Sex with FUCKBUDDY partners** (among HIV negative)				
(Last 6 months)	266 (9.4%)	245 (11.3%)		
Condoms only	264 (9.3%)	25 (1.1%)	7 (2.8%)	14 (3.4%)
PrEP	123 (4.3%)	119 (5.5%)	0 (0%)	239 57.6%
Insertive condomless—no PrEP	108 (3.8%)	86 (4.0%)	4 (1.6%)	0 (0%)
Receptive condomless withdrawal—no PrEP	206 (7.3%)	126 (5.8%)	22 (8.7%)	0 (0%)
Receptive condomless ejaculation–no PrEP			80 (31.6%)	0 (0%)
**Sex with FUCKBUDDY partners** (among HIV positive)				
(Last 6 months)	8 (0.3%)			
Condoms only	26 (0.9%)	8 (0.4%)	0 (0%)	0 (0%)
Undetectable and treated	41 (1.4%)	26 (1.2%)	0 (0%)	0 (0%)
Receptive condomless only–no ARV	1 (0.0%)	41 (1.9%)	0 (0%)	0 (0%)
Insertive comdomless withdrawal–no ARV	3 (0.1%)	0 (0%)	1 (0.4%)	0 (0%)
Insertive condomless ejaculation–no ARV		1 (0.0%)	2 (0.8%)	0 (0%)
**Sex with FUCKBUDDY partners** (among HIV unknown)				
(Last 6 months)		35 (1.6%)		
Condoms only	39 (1.4%)	9 (0.4%)	3 (1.2%)	1 (0.2%)
Insertive condomless—no PrEP	20 (0.7%)	11 (0.5%)	10 (4.0%)	1 (0.2%)
Receptive condomless withdrawal—no PrEP	20 (0.7%)	11 (0.5%)	8 (3.2%)	1 (0.2%)
Receptive condomless ejaculation–no PrEP	30 (1.1%)		15 (5.9%)	4 (1.0%)

Three types of sexual partners were described and assessed: boyfriends (regular partner with an ongoing, usually romantic, relationship), ‘fuckbuddies’ (regular partners with whom one is not in a committed relationship), and casual partners (all other non-regular partners) [31]. Sexual behaviour in the previous 6 months was reported. GBM reported if they ‘never’, ‘occasionally’, or ‘often’ engaged in specific anal sex practices and positioning with each partner type (insertive, receptive), condom use, and whether they ejaculated during condomless anal intercourse. Drug use and frequency of use were also measured. The list of drugs measured can be found in Table 1. Participants were asked if they used the drugs at the following frequencies: never, over 6 months ago, in the past 6 months, monthly, and weekly. For the analysis, ‘never’ and ‘over 6 months ago’ were merged, as were ‘monthly’ and ‘weekly’ use, resulting in three categories.

Measures were selected from the survey based on the literature identifying relationships between GBM’s sexual behavior, HIV risk, and use of HIV prevention practices [32–33]. Social engagement with gay men was assessed using a scale created from two items (the proportion of friends who are gay men, and amount of free time spent with gay men), higher scores indicated greater social engagement [32]. The proportion of gay friends was scored from ‘none’ (0) to ‘all’ (4). The amount of free time spent with gay men was scored from ‘none’ (0) to ‘a lot’ (3). These items were added together to create the social engagement measure (scored from 0 to 7). The Kalichman and Rompa measure of sexual sensation-seeking was also included [33]. A five-point scale was used to measure the degree to which participants identified with being a top or a bottom during anal sex, from “very much a bottom” (1) to “very much a top” (5).

Considering that multi-level approaches are needed to develop effective HIV prevention responses, the measures selected in this analysis range from the individual, interpersonal and community levels of the modified social ecological model [29,34]. Factors at the public policy level are also considered and discussed in this paper.

### Analysis

**Fig 1:**
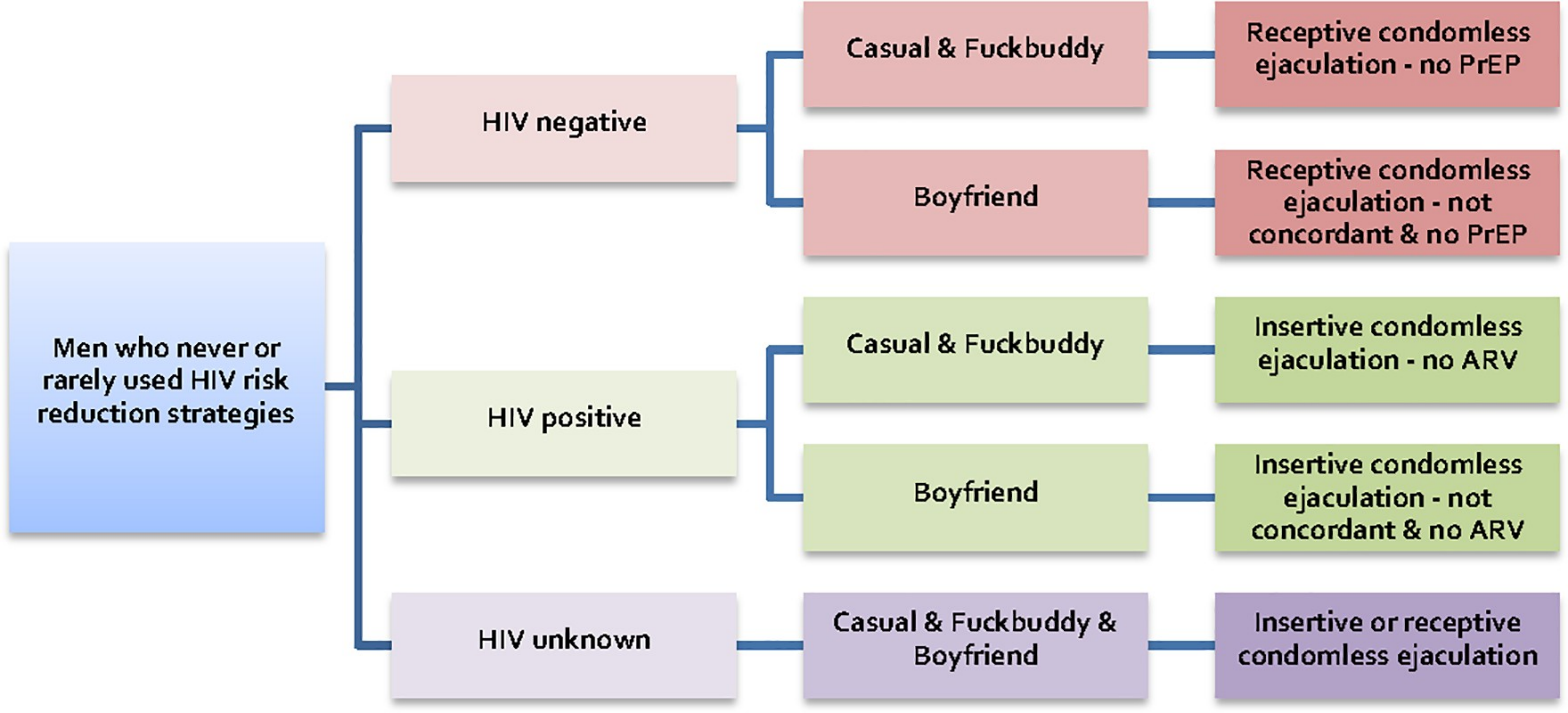
Definition of No Risk Reduction (NRR) men. The category of men who never or rarely used HIV risk reduction strategies (NRR men).

This analysis used baseline data and was conducted using *STATA*, *version 14*, software. The category of “men who never or rarely used HIV risk reduction strategies” (NRR men) was constructed according to the following criteria (Fig 1). HIV-negative men not using PrEP were included in the NRR group if they had frequently engaged in receptive condomless anal intercourse with ejaculation with casual partners, fuckbuddies, or boyfriends who were either HIV-positive and not on treatment, or of unknown HIV status. HIV-positive men who were not on ART were included in the NRR group if they had engaged in insertive condomless anal intercourse with ejaculation with casual partners, fuckbuddies, or boyfriends who were HIV-negative or of unknown HIV status. Men of unknown HIV status were included in the NRR group if they had engaged in any insertive or receptive condomless anal intercourse with ejaculation with any partner type.

NRR men were compared with two groups: 1) men who reported using PrEP and 2) men who reported the frequent use of risk reduction strategies other than PrEP (known hereafter as FRR men). The use of each strategy (condoms, strategic positioning, withdrawal, TasP) was measured from ‘none (1)’ to ‘always (5)’ on a 5-point scale. GBM who reported often or always using a strategy were classified as frequently using it. The strategy most commonly used by this group was condoms for anal intercourse.

Categorical variables were analyzed using Pearson’s chi-square test and t-tests for continuous variables. We used Type I error of 5% for these analyses. To assess statistical associations with having used little to no risk reduction strategies, we used logistic regression models. Items in our bivariate analyses included: age, education, ethnicity, sexual orientation, sexual sensation seeking, gay social engagement, use of amyl nitrite, gamma-hydroxybutyrate (GHB), cocaine, crystal methamphetamine or erectile dysfunction medication (EDM) in the previous six months, and preference for the top (insertive) or bottom (receptive) position during anal sex. Associations with a p-value of less than 0.05 in bivariate analyses were included in the multivariate analyses. We then calculated adjusted odds ratios (AOR) and 95% confidence intervals (CI).

## Results

### Sample demographics

Participant characteristics are shown in Table 1. The mean age of the 2,920 men in this sample was 35 years (S.D. 13.0). The majority (72%) identified as Anglo-Australian and had a university degree (57%). Most men (n = 2,609, 89%) identified as gay or homosexual, 250 men identified as bisexual (9%) and a smaller proportion of men identified otherwise (n = 57, 2%). There were 31 men who identified as transgender in the sample. One in seven men (n = 429, 15%) had not been tested for HIV or did not know their HIV status, 196 men (7%) reported being HIV-positive and 2,295 (79%) reported that they were HIV-negative. Overall, there were 2,252 FRR men (77%), 415 men on PrEP (14%) and 253 NRR men (9%). Only 86 men with unknown HIV status (3% of the entire sample) and 6 HIV-positive men (0.2% of the entire sample) were in the NRR category; most were HIV-negative men.

### NRR versus men on PrEP

NRR men were similar to PrEP users in terms of ethnicity, sexual orientation, use of GHB or crystal methamphetamine, and preferred position in anal sex (Table 2). In the multivariate analysis, NRR men were younger than men on PrEP (aOR = 0.96; 95% CI: 0.95 to 0.98; p<0.0001), less socially engaged with gay men (aOR = 0.75; 95% CI: 0.66 to 0.84; p<0.0001) and were less likely to have completed a postgraduate (aOR = 0.47; 95% CI: 0.29 to 0.76; p<0.05) or undergraduate degree (aOR = 0.51; 95% CI: 0.34 to 0.78; p<0.05). The NRR men were less likely than PrEP users to have recently used amyl nitrite (aOR = 0.63; 95% CI: 0.41 to 0.96; p<0.05), EDM (aOR = 0.53; 95% CI: 0.31 to 0.89; p<0.05) and cocaine (aOR = 0.11; 95% CI: 0.01 to 0.85; p<0.05).

**Table 2:** Logistic regression analysis of No Risk Reduction (NRR) GBM versus PrEP users.

	NRR (n = 253)	PrEP (n = 415)	Bivariate OR (95% CI)	Multivariate AOR (95% CI)
**Age (M, SD)**	32.4 (13.3)	41.2 (11.8)	0.94 (0.93 to 0.96)***	0.96 (0.95 to 0.98)***
**Education (M, SD)**				
Less than undergraduate	151 (59.7%)	143 (34.5%)	1.00	1.00
Undergraduate	62 (24.5%)	139 (33.5%)	0.42 (0.29 to 0.62)***	0.51 (0.34 to 0.78)*
Postgraduate	40 (15.8%)	133 (32.0%)	0.28 (0.19 to 0.43)***	0.47 (0.29 to 0.76)*
**Ethnicity/Cultural**				
**Background**	71 (28.4%)			
Other Background	179 (71.6%)	123 (29.6%)	1.00	
Anglo-Australian		292 (70.4%)	1.06 (0.75 to 1.50)	
**Sexual orientation**				
Gay	224 (88.5%)	391 (94.2%)	1.00	1.00
Bisexual / Other	29 (11.5%)	24 (5.8%)	2.11 (1.20 to 3.71)*	1.49 (0.72 to 3.12)
**Sexual Sensation**				
**Seeking (M, SD)**	32.4 (6.3)	32.7 (5.6)	0.99 (0.96 to 1.02)	
**Gay social**				
**engagement (M, SD)**	3.4 (1.6)	4.4 (1.5)	0.66 (0.59 to 0.73)***	0.75 (0.66 to 0.84)***
**Amyl nitrite use**				
Never used & over 6M	147 (58.1%)	142 (34.2%)	1.00	1.00
Past 6 Months	42 (16.6%)	89 (21.4%)	0.46 (0.30 to 0.70)***	0.60 (0.36 to 0.99)*
Weekly/Monthly	64 (25.3%)	184 (44.3%)	0.34 (0.23 to 0.48)***	0.63 (0.41 to 0.96)*
**Cocaine use**				
Never used & over 6M	224 (88.5%)	299 (72.0%)	1.00	1.00
Past 6 Months	28 (11.1%)	96 (23.1%)	0.39 (0.25 to 0.61)***	0.52 (0.31 to 0.87)*
Weekly/Monthly	1 (0.4%)	20 (4.8%)	0.07 (0.01 to 0.50)*	0.11 (0.01 to 0.85)*
**EDM use**				
Never used & over 6M	195 (77.1%)	188 (45.3%)	1.00	1.00
Past 6 Months	28 (11.1%)	93 (22.4%)	0.29 (0.18 to 0.46)***	0.55 (0.33 to 0.92)*
Weekly/Monthly	30 (11.9%)	134 (32.3%)	0.22 (0.14 to 0.34)***	0.53 (0.31 to 0.89)*
**GHB use**				
Never used & over 6M	233 (92.1%)	322 (77.6%)	1.00	1.00
Past 6 Months	12 (4.7%)	53 (12.8%)	0.31 (0.16 to 0.60)***	0.62 (0.25 to 1.50)
Weekly/Monthly	8 (3.2%)	40 (9.6%)	0.28 (0.13 to 0.60)**	0.82 (0.29 to 2.33)
**Crystal Meth use**				
Never used & over 6M	211 (83.4%)	318 (76.6%)	1.00	
Past 6 Months	25 (9.9%)	50 (12.0%)	0.75 (0.45 to 1.26)	
Weekly/Monthly	17 (6.7%)	47 (11.3%)	0.55 (0.30 to 0.97)	
**Top versus Bottom**				
**Identity**	5 (2.0%)	21 (5.1%)	1.00	1.00
Very much a top	32 (12.7%)	72 (17.3%)	1.87 (0.65 to 5.39)	2.71 (0.63 to 11.64)
Top	114 (45.4%)	196 (47.2%)	2.44 (0.90 to 6.66)	3.30 (0.82 to 13.27)
Versatile	75 (29.9%)	96 (23.1%)	3.28 (1.18 to 9.12)*	4.35 (1.05 to 18.01)
Bottom	25 (10.0%)	30 (7.2%)	3.50 (1.15 to 10.6)*	2.59 (0.57 to 11.77)
Very much a bottom				

*P<0.05.

**P<0.001.

***P<0.0001.

### NRR versus FRR

NRR and FRR men were similar in terms of ethnicity, sexual orientation, social engagement with gay men and recent drug use (Table 3). In the multivariate analysis, NRR men were similar in age and less likely to have completed a postgraduate (aOR = 0.46; 95% CI: 0.31 to 0.67; p<0.0001) or undergraduate degree (aOR = 0.59; 95% CI: 0.43 to 0.83; p<0.05) than FRR men. NRR men scored higher on the sexual sensation-seeking scale than FRR men (aOR = 1.09; 95% CI: 1.06 to 1.12; p<0.0001) and were more likely than FRR men to identify as versatile (aOR = 2.94; 95% CI: 1.05 to 8.23; p<0.05), a bottom (aOR = 3.40; 95% CI: 1.20 to 9.63; p<0.05) or very much a bottom for anal sex (aOR = 3.73; 95% CI: 1.21 to 11.6; p<0.05).

**Table 3:** Logistic regression analysis of No Risk Reduction (NRR) GBM versus Frequent Risk Reduction (FRR) GBM.

	NRR (n = 253)	FRR (n = 2,252)	Bivariate OR (95% CI)	Multivariate AOR (95% CI)
**Age (M, SD)**	32.4 (13.3)	34.7 (12.9)	0.99 (0.98 to 1.00)*	0.99 (0.98 to 1.00)
**Education (M, SD)**		970 (43.2%)		
Less than undergrad	151 (59.7%)	714 (31.8%)	1.00	1.00
Undergraduate	62 (24.5%)	562 (25.0%)	0.56 (0.41 to 0.76)***	0.59 (0.43 to 0.83)*
Postgraduate	40 (15.8%)		0.46 (0.32 to 0.66)***	0.46 (0.31 to 0.67)***
**Ethnicity/Cultural**				
**Background**	71 (28.4%)	604 (27.0%)		
Other background	179 (71.6%)	1,637 (73.0%)	1.00	
Anglo-Australian			0.93 (0.70 to 1.24)	
**Sexual orientation**				
Gay	224 (88.5%)	1,994 (88.5%)	1.00	
Bisexual / Other	29 (11.5%)	258 (11.5%)	1.00 (0.67 to 1.50)	
**Sexual Sensation**				
**Seeking (M, SD)**	32.4 (6.3)	29.1 (6.2)	1.09 (1.07 to 1.12)***	1.09 (1.06 to 1.12)***
**Gay social**				
**engagement (M, SD)**	3.4 (1.6)	3.5 (1.6)	0.97 (0.89 to 1.05)	
**Amyl nitrite use**				
Never used & over 6M	147 (58.1%)	1,517 (67.4%)	1.00	1.00
Past 6 Months	42 (16.6%)	367 (16.3%)	1.18 (0.82 to 1.70)	0.99 (0.65 to 1.51)
Weekly/Monthly	64 (25.3%)	368 (16.3%)	1.79 (1.31 to 2.46)***	1.30 (0.89 to 1.91)
**Cocaine use**				
Never used & over 6M	224 (88.5%)	1,957 (86.9%)	1.00	
Past 6 Months	28 (11.1%)	247 (11.0%)	0.99 (0.65 to 1.50)	
Weekly/Monthly	1 (0.4%)	48 (2.1%)	0.18 (0.03 to 1.33)	
**EDM use**				
Never used & over 6M	195 (77.1%)	1,730 (76.8%)	1.00	
Past 6 Months	28 (11.1%)	291 (12.9%)	0.85 (0.56 to 1.29)	
Weekly/Monthly	30 (11.9%)	231 (10.3%)	1.15 (0.77 to 1.73)	
**GHB use**				
Never used & over 6M	233 (92.1%)	2,099 (93.2%)	1.00	
Past 6 Months	12 (4.7%)	114 (5.1%)	0.95 (0.52 to 1.75)	
Weekly/Monthly	8 (3.2%)	39 (1.7%)	1.85 (0.85 to 4.00)	
**Meth use**				
Never used & over 6M	211 (83.4%)	2,010 (89.3%)	1.00	1.00
Past 6 Months	25 (9.9%)	135 (6.0%)	1.76 (1.13 to 2.77)*	1.31 (0.64 to 2.70)
Weekly/Monthly	17 (6.7%)	107 (4.8%)	1.51 (0.89 to 2.57)	0.72 (0.37 to 1.38)
**Top versus Bottom**	5 (2.0%)	11 (0.5%)		
**Identity**	32 (12.7%)	426 (19.0%)	1.00	1.00
Very much a top	114 (45.4%)	1,056 (47.0%)	1.67 (0.64 to 4.38)	2.06 (0.70 to 6.05)
Top	75 (29.9%)	532 (24.4%)	2.40 (0.96 to 5.99)	2.94 (1.05 to 8.23)*
Versatile	25 (10.0%)	120 (5.3%)	3.13 (1.24 to 7.92)*	3.40 (1.20 to 9.63)*
Bottom			4.63 (1.71 to 12.5)*	3.73 (1.21 to 11.6)*
Very much a bottom				

*P<0.05.

**P<0.001.

***P<0.0001.

## Discussion

We found that while most Australian GBM (91%) used some form of HIV risk reduction, nearly one in ten (9%) rarely used an HIV risk reduction strategy. There were fewer differences between the NRR men and the FRR men (men who reported the frequent use of risk reduction strategies other than PrEP) than between the NRR men and the men on PrEP. Whether this reflects issues that NRR men have with condoms, the primary strategy of FRR men, requires further exploration. Most HIV-positive men in Australia are on ART with undetectable viral loads [22–23]. It is therefore not surprising that very few of the NRR men were HIV-positive (2.4%).

While NRR men scored higher on sexual sensation seeking than FRR men, they did not score higher than men who used PrEP. Therefore, the NRR men and the men who used PrEP appeared similar both behaviorally and in terms of their sexual desires. GBM who scored higher on the sexual sensation seeking scale tended to be more sexually adventurous and were more likely to engage in HIV risk behavior [33,35–36]. FRR men may be less inclined to engage in riskier sexual practices in general and may be more comfortable practicing a HIV risk reduction strategy like condoms. PrEP could be explored as a priority strategy for uptake among NRR men particularly when considering that NRR men scored higher on the sexual sensation seeking scale and were more likely to identify as bottoms than FRR men. Risk reduction strategies, condoms in particular, may interfere with the sex practices preferred by NRR men. PrEP may provide a way for these men to reduce risk without necessarily compromising their sexual desires. By reducing anxiety regarding anal sex, PrEP may also be a more palatable strategy for NRR men.

Levels of education among NRR men were significantly lower than both FRR men and men on PrEP. In the US lower levels of education have been associated with lower levels of HIV prevention knowledge [37]. The NRR men were also less socially engaged with other gay men than the GBM using PrEP. Lower levels of social connectedness to other gay men may reduce opportunities to hear about risk reduction strategies, including PrEP, from peers. Social networks have been associated with increases in PrEP uptake [28,38]. NRR men may be generally less exposed to the diffusion of knowledge and innovation among GBM in Australia. Within particular networks of GBM biomedical forms of HIV prevention, such as PrEP, are increasingly being utilized, leading to increasing/greater normalisation in practice [26]. GBM using PrEP were also significantly older than NRR men. Older GBM may be more confident about their sexuality and more connected to gay community than younger GBM [32]. In San Francisco where PrEP has been available since 2012, older GBM have also been shown to be more likely to adopt PrEP [39].

This study has limitations. The analysis is based on self-report data. As such, the data may be subject to desirability bias and it is possible that participants may have underreported experiences of not using a risk reduction strategy. The analyses presented here were cross sectional, using baseline data only. Future longitudinal analyses will allow us to monitor changes in the use of HIV risk reduction strategies over time. Those types of analyses would also help to understand the factors that may influence the uptake of strategies. Such research is needed to better craft meaningful health promotion interventions with this population. This analysis was unable to account for serosorting among casual partners or fuckbuddies, as this was not measured. Serosorting is likely to have been practiced by at least some of the NRR men and other participants [6], despite it generally being viewed as an unreliable risk reduction strategy [40]. Overall the NRR men in our analysis were similar to other GBM in our sample.

This analysis demonstrates the need for further interventions with and for GBM in Australia at the individual, interpersonal and community levels [29,34]. Continuing efforts to engage GBM who are less socially connected, those who are younger and GBM who are less educated about their sexual health, and about PrEP and condom use specifically, are required to ensure that equitable access to appropriate prevention technologies and information is achieved. Health promotion work with GBM who are more sexually adventurous may also increase the use of HIV risk reduction strategies among higher risk men. The increased availability of PrEP in Australia, due to its recent listing on the Pharmaceutical Benefits Scheme [16], provides further opportunity for NRR men to engage with this strategy. The mobilization of non-gay community partners, such as general practitioners, may be essential to ensure that men who are less socially connected to other GBM have greater access to PrEP. However, PrEP can only reduce risk in relation to HIV; the risk of other STIs is unaffected by use of PrEP alone. Having the means to obtain PrEP and the understanding of how and why to use it are critical to its uptake. More information about the health literacy of men who do not frequently use HIV risk reduction should be explored. The adoption of a HIV risk reduction strategy could be part of a broader upskilling in sexual health knowledge for this group. This should also include improving the uptake of HIV testing for this group.

## Conclusion

Although most GBM use some form of HIV risk reduction, a minority of men rarely or never use any form of risk reduction and remain at high risk of HIV infection, despite the growing and widespread availability of PrEP. While PrEP may be an appropriate tool for NRR men, we need to better understand why they do not use it. In our sample, younger, less educated and less socially engaged gay men were less likely to use any risk reduction strategies. These men may have less exposure to information about HIV risk reduction and sexual health promotion. Efforts to engage GBM who are less socially connected to gay community are warranted to encourage them to use an effective form of HIV risk reduction.

## References

[1] Kirby Institute: HIV, viral hepatitis and sexually transmissible infections in Australia: annual surveillance report 2017. Sydney: Kirby Institute, UNSW Sydney; 2017.

[2] Jin F, Crawford J, Prestage GP, et al: Unprotected anal intercourse, risk reduction behaviours, and subsequent HIV infection in a cohort of homosexual men. AIDS. 2009; 23(2): 243–52. 19098494 10.1097/QAD.0b013e32831fb51a PMC2768371

[3] Kennedy CE, Bernard LJ, Muessig KE, et al: Serosorting and HIV/STI infection among HIV-negative MSM and transgender people: a systematic review and meta-analysis to inform WHO guidelines. Journal of sexually transmitted diseases. 2013; 2013. 10.1155/2013/583627 PMC4437432 26316960

[4] Fonner VA, Dalglish SL, Kennedy CE, et al: Effectiveness and safety of oral HIV pre-exposure prophylaxis (PrEP) for all populations: A systematic review and meta-analysis. AIDS (London, England). 7 2016; 30 (12):1973–83. 10.1097/QAD.0000000000001145 PMC4949005 27149090

[5] Cohen MS, Chen YQ, McCauley M, et al: Prevention of HIV-1 infection with early antiretroviral therapy. New England journal of medicine. 2011; 365(6):493–505. 21767103 10.1056/NEJMoa1105243 PMC3200068

[6] Holt M, Lea T, Mao L, et al: HIV prevention by Australian gay and bisexual men with casual partners: the emergence of undetectable viral load as one of a range of risk reduction strategies. Journal Acquired Immune Deficiency Syndromes (JAIDS), 2015 12 15;70(5):545–8. 10.1097/QAI.0000000000000787 26258572

[7] ACON: You Choose Campaign. 2017 https://endinghiv.org.au/stay-safe/

[8] Department of Health: Eighth National HIV Strategy 2018–2022. Commonwealth of Australia 2018 http://www.health.gov.au/internet/main/publishing.nsf/Content/ohp-bbvs-1/$File/HIV-Eight-Nat-Strategy-2018-22.pdf

[9] NSW Health: NSW HIV Strategy 2016–2020. Sydney: NSW Ministry of Health 2015 https://www.health.nsw.gov.au/endinghiv/Publications/nsw-hiv-strategy-2016-2020.pdf

[10] Haire B: Reciprocity and citizenship in the adoption of a new HIV prevention technology. Seminar Kirby Institute Tuesday, 17 October 2017, https://kirby.unsw.edu.au/event/dr-bridget-haire-reciprocity-and-citizenship-adoption-new-hiv-prevention-technology

[11] Race K: Framing responsibility. Journal of bioethical inquiry. 2012 9 1;9(3):327–38. 10.1007/s11673-012-9375-x 23180333

[12] Mao L, Holt M, Newman C, et al: Annual Report of Trends in Behaviour 2017: HIV and STIs in Australia. 2017; Sydney: Centre for Social Research in Health, UNSW Sydney 10.4225/53/59faa5c891779

[13] Grant RM, Lama JR, Anderson PL, et al : Preexposure chemoprophylaxis for HIV prevention in men who have sex with men. New England Journal of Medicine. 2010 12 30; 363(27):2587–99. 21091279 10.1056/NEJMoa1011205 PMC3079639

[14] Ryan KE, Mak A, Stoove M, et al: Protocol for an HIV pre-exposure prophylaxis (PrEP) population level intervention study in Victoria Australia: the PrEPX study. Frontiers in Public Health. 2018;6(151). 10.3389/fpubh.2018.00151 PMC5987055 29896468

[15] Zablotska IB, Selvey C, Guy R, et al: Expanded HIV pre-exposure prophylaxis (PrEP) implementation in communities in New South Wales, Australia (EPIC-NSW): design of an open label, single arm implementation trial. BMC Public Health. 2018;18(1):210. 29394918 10.1186/s12889-017-5018-9 PMC5797394

[16] The Pharmaceutical Benefits Scheme (PBS): Tenofovir with emtricitabine: Tablet containing tenofovir disoproxil maleate 300 mg with emtricitabine 200 mg. Available online at https://www.pbs.gov.au/medicine/item/11149T-11296M

[17] Kippax S, Connell RW, Dowsett GW, et al: Sustaining safe sex. AIDS.1993; 7(2):257–64. 8466689

[18] Le Talec J, Jablonski O: Seroadaptation instead of serosorting: a broader concept and a more precise process model. In: XVII International AIDS Conference Mexico City 2008.

[19] Jin F, Jansson J, Law M, et al: Per-contact probability of HIV transmission in homosexual men in Sydney in the era of HAART. AIDS (London, England), 2010, 24(6), 907–13. 10.1097/QAD.0b013e3283372d90 PMC2852627 20139750

[20] Down I, Ellard J, Bavinton BR, et al: In Australia, most HIV infections among gay and bisexual men are attributable to sex with ‘new’partners. AIDS and Behavior. 2017 8 1;21(8):2543–50. 28283774 10.1007/s10461-017-1747-0

[21] Gray RT, Wilson DP, Guy RJ, et al: Undiagnosed HIV infections among gay and bisexual men increasingly contribute to new infections in Australia. Journal of the International AIDS Society. 2018 4;21(4):e25104. 29638044 10.1002/jia2.25104 PMC5894250

[22] Holt M, Lea T, Mao L, et al: Community-level changes in condom use and uptake of HIV pre-exposure prophylaxis by gay and bisexual men in Melbourne and Sydney, Australia: results of repeated behavioural surveillance in 2013–17. The Lancet HIV. 2018 8 1;5(8):e448–56. 10.1016/S2352-3018(18)30072-9 29885813

[23] Mao L, Holt M, Newman C, et al: Annual Report of Trends in Behaviour 2018: HIV and STIs in Australia. 2018, Sydney: Centre for Social Research in Health, UNSW Sydney 10.26190/5b7a3caa2a994

[24] Lea T, Kolstee J, Murphy D, et al: Changing attitudes to and engagement with biomedical HIV prevention by gay and bisexual men: key findings from the PrEPARE Project 2017. 2017, Sydney: Centre for Social Research in Health, UNSW Sydney 10.4225/53/5a791a59a65e4

[25] Prestage G, Degenhardt L, Jin F, et al: Predictors of frequent use of amphetamine type stimulants among HIV-negative gay men in Sydney, Australia. Drug and Alcohol Dependence. 2007 12 1;91(2–3):260–8. 17640831 10.1016/j.drugalcdep.2007.06.009 PMC2699371

[26] Hammoud MA, Vaccher S, Jin F, et al: The new MTV generation: Using methamphetamine, Truvada™, and Viagra™ to enhance sex and stay safe. International Journal of Drug Policy. 2018 5 1;55:197–204. 10.1016/j.drugpo.2018.02.021 29526546

[27] Holt M, Murphy DA: Individual versus community-level risk compensation following preexposure prophylaxis of HIV. American journal of public health. 2017 10;107(10):1568–71. 10.2105/AJPH.2017.303930 28817332 PMC5607662

[28] Holt M, Lea T, Bear B, et al: Trends in Attitudes to and the Use of HIV Pre-exposure Prophylaxis by Australian Gay and Bisexual Men, 2011–2017: Implications for Further Implementation from a Diffusion of Innovations Perspective. AIDS and Behavior. 2018 12 11:1–2. 10.1007/s10461-018-2368-y 30539496

[29] Kaufman MR, Cornish F, Zimmerman RS, et al: Johnson BT. Health behavior change models for HIV prevention and AIDS care: practical recommendations for a multi-level approach. Journal of acquired immune deficiency syndromes (1999). 2014 8 15;66(Suppl 3):S250. 25007194 10.1097/QAI.0000000000000236 PMC4536982

[30] Hammoud MA, Jin F, Degenhardt L, et al: Following Lives Undergoing Change (Flux) study: implementation and baseline prevalence of drug use in an online cohort study of gay and bisexual men in Australia. International Journal of Drug Policy. 2017 3 1;41:41–50. 28081482 10.1016/j.drugpo.2016.11.012

[31] Bavinton BR, Duncan D, Grierson J, et al: The meaning of ‘regular partner’ in HIV research among gay and bisexual men: implications of an Australian cross-sectional survey. AIDS and Behavior. 2016;20(8):1777–84. 26971284 10.1007/s10461-016-1354-5

[32] Zablotska IB, Holt M, & Prestage G: Changes in gay men’s participation in gay community life: implications for HIV surveillance and research. AIDS and Behavior, 2012; 16(3), 669–675. 21424273 10.1007/s10461-011-9919-9

[33] Kalichman SC, & Rompa D: Sexual sensation seeking and sexual compulsivity scales: Validity, and predicting HIV risk behavior. Journal of Personality Assessment, 1995; 65(3), 586–601. 8609589 10.1207/s15327752jpa6503_16

[34] Baral S, Logie CH, Grosso A, et al: Modified social ecological model: a tool to guide the assessment of the risks and risk contexts of HIV epidemics. BMC public health. 2013 12;13(1):482. 23679953 10.1186/1471-2458-13-482 PMC3674938

[35] Difranceisco W, Ostrow DG, Chmiel JS: Sexual adventurism, high-risk behavior, and human immunodeficiency virus-1 seroconversion among the Chicago MACS-CCS cohort, 1984 to 1992: a case-control study. Sexually transmitted diseases. 1996 11 1;23(6):453–60. 8946628 10.1097/00007435-199611000-00003

[36] Crawford I, Hammack PL, McKirnan DJ, et al: Sexual sensation seeking, reduced concern about HIV and sexual risk behaviour among gay men in primary relationships. AIDS care. 2003 8 1;15(4):513–24. 14509866 10.1080/0954012031000134755

[37] Sharma A, Sullivan SP, Stephenson RB.: Detailed knowledge about HIV epidemiology and transmission dynamics and their associations with preventive and risk behaviors among gay, bisexual, and other men who have sex with men in the United States. JMIR public health and surveillance. 2017 1;3(1). 10.2196/publichealth.7255 PMC5359415 28264795

[38] Hammoud MA, Vaccher S, Jin F, et al: HIV pre-exposure prophylaxis (PrEP) uptake among gay and bisexual men in Australia and factors associated with the non-use of PrEP among eligible men: Results from a prospective cohort study. J Acquir Immune Defic Syndr. 2019;Publish Ahead of Print. 10.1097/QAI.0000000000002047 30973548

[39] Snowden JM, Chen YH, McFarland W, et al: Prevalence and characteristics of users of pre-exposure prophylaxis (PrEP) among men who have sex with men, San Francisco, 2014 in a cross-sectional survey: implications for disparities. Sex Transm Infect. 2017 2 1;93(1):52–5. 27356041 10.1136/sextrans-2015-052382 PMC5241245

[40] Holt M, Draper BL, Pedrana AE, et al: Comfort Relying on HIV Pre-exposure Prophylaxis and Treatment as Prevention for Condomless Sex: Results of an Online Survey of Australian Gay and Bisexual Men. AIDS and Behavior. 2018 3 21:1–0. 10.1007/s10461-018-2097-2 29564695

